# AI-driven tools for the prediction of obesity-related vascular diseases: stakeholder perspectives and challenges

**DOI:** 10.3389/fpubh.2025.1713882

**Published:** 2025-12-04

**Authors:** Kaatje Goossens, Pascal Borry, Tessa Marie Forehand, Eva Van Steijvoort

**Affiliations:** Centre for Biomedical Ethics and Law, Department of Public Health and Primary Care, KU Leuven, Leuven, Belgium

**Keywords:** artificial intelligence, cardiovascular risk prediction, obesity, stakeholder perspectives, development, implementation

## Abstract

**Introduction:**

Within the Horizon Europe-funded AI-POD (AI-based tools for the Prediction of Obesity-related vascular Diseases) project, a clinical decision support system and citizen-facing mobile health application are being developed to enable personalized cardiovascular risk prediction in individuals living with obesity, through the integration of clinical, imaging, laboratory and lifestyle data. To inform the responsible development and implementation of these innovations, this study explored stakeholder perspectives on anticipated benefits, concerns, and challenges across four European countries.

**Methods:**

Semi-structured interviews were conducted with 21 stakeholders between February and July 2025. Participants represented diverse (professional) backgrounds including radiology (*n* = 5), artificial intelligence (*n* = 4), medical informatics and healthcare innovation (*n* = 2), dietetics (*n* = 2), endocrinology (*n* = 2), and general practice (*n* = 1). In addition, our sample included two patient representatives (*n* = 2), as well as individuals with expertise in social sciences and ethics (*n* = 1), law and policy (*n* = 1), and public health (*n* = 1). Most were based in Belgium (*n* = 16), with others from Austria (*n* = 3), the United Kingdom (*n* = 1), and Sweden (*n* = 1). Seven participants were affiliated with the AI-POD consortium, while 14 were external experts. All interviews were audio-recorded, transcribed verbatim, and analyzed using inductive content analysis.

**Results:**

Participants identified several benefits of the AI-POD tools, including the integration of multimodal data, improved risk stratification, and enhanced patient engagement and health literacy. However, concerns were raised about potential anxiety stemming from risk scores, the reinforcement of weight stigma, limited evidence supporting personalized lifestyle recommendations, and equitable access to the tools. Key challenges included data heterogeneity, algorithmic bias, small sample sizes, and technological barriers such as device incompatibility and varying levels of digital literacy. Participants anticipated that implementation would be further complicated by difficulties in engaging patients and by healthcare professionals’ reluctance to adopt solutions that fall outside established guidelines.

**Conclusion:**

While stakeholders acknowledged the promise of the AI-POD tools for advancing personalized cardiovascular risk prediction in individuals living with obesity, they also identified critical challenges related to equitable access, sustained user engagement, and effective integration into clinical practice. Addressing these challenges will be essential for the successful implementation, adoption, and uptake of the tools envisioned within the AI-POD project.

## 1 Introduction

Artificial intelligence (AI) is defined as the ability of algorithms encoded in technology to learn from data so that they can perform automated tasks without each step in the process having to be explicitly programmed by humans (1, 2). In healthcare and public health, AI technologies offer transformative potential to enhance clinical decision-making, improve diagnostic accuracy, reduce human error, and support efficient allocation of resources (3–5). Beyond clinical contexts, AI applications are increasingly designed to empower patients through self-management tools that enable personalized monitoring and lifestyle interventions related to nutrition, physical activity, and disease risk (1, 6).

Realizing the potential of AI, however, depends not only on technological performance but also on how these systems are perceived and adopted by stakeholders. Understanding these perspectives is important, as they highlight not only the perceived benefits of AI but also the concerns that may limit its uptake. Studies with patients and members of the general population have shown that, while many recognize the potential of AI to improve diagnostics and treatment support, they also express concerns related to bias, reduced clinician–patient communication, lack of trust in algorithmic processes, and insufficient regulatory or liability frameworks (7, 8). Esmaeilzadeh et al. recognized that technological issues, particularly those related to performance and communication, emerged as the strongest predictors of perceived risk of AI tools (7). These findings align with previous research indicating that uncertainty about how AI systems function is a key factor influencing perceptions of risk associated with their use in healthcare (9). Similar concerns have emerged among healthcare professionals evaluating AI tools in cardiovascular medicine. For instance, in a study of an AI-driven blood utilization calculator, clinicians generally regarded the tool as user-friendly and valuable for efficiency and patient outcomes, yet adoption was tempered by concerns regarding risk, accountability, and trust (10). Comparable themes emerged in a qualitative evaluation of QRhythm, an AI-based decision support system for atrial fibrillation rhythm management (11). Here, providers emphasized the safety of recommendations as the highest priority, followed by clinical integrity, algorithmic transparency, and understanding of the population used to train the model (11). Although the tool was perceived as easy to use, variable confidence in its recommendations highlighted the importance of reliability and interpretability for successful implementation (11). Collectively, these findings underscore that safety, interpretability and trustworthiness are pivotal for clinician acceptance of AI-based decision support.

In the field of obesity care, AI has been associated with several anticipated benefits, including improved personalization through real-time data integration, greater precision in risk stratification, and potential cost-effectiveness through optimized resource allocation (12). Nevertheless, challenges remain (12). Teke et al. noted issues such as sustaining user engagement over time, and the risk that algorithmic bias could exacerbate existing health disparities (12).

In addition to individual factors, organizational and systemic elements may also influence AI implementation. A qualitative study in Sweden identified barriers spanning external regulatory and policy conditions, internal capacity for strategic change management, and broader transformations in healthcare professions and practices (13). The study argued that effective implementation requires cross-sectional strategies to build AI-specific capacity, supported by robust legal and policy frameworks. Participants in this study viewed sustained investments of time and resources, coupled with collaboration among healthcare organizations, local authorities, and industry stakeholders, as essential for enabling large-scale AI integration (13).

The Horizon Europe-funded AI-POD (AI-based tools for the Prediction of Obesity-related vascular Diseases) project builds on these insights by developing and validating trustworthy AI-based tools for assessing and predicting the risk of cardiovascular diseases and related complications in individuals living with obesity. The project’s central innovation is an AI-based risk score that integrates clinical, laboratory, imaging and continuous lifestyle data – including activity, heart rate, and diet – to enhance cardiovascular risk prediction. This score will be made available to patients through a citizen-facing mobile health application, designed to support personalized, self-directed health management and ongoing risk monitoring. Complementing this, a clinical decision support system will be developed for use by healthcare providers to deliver risk assessments that inform prevention, management, and treatment strategies in obesity care (14).

While existing studies on stakeholder perspectives toward specific AI-tools have predominantly been conducted in the U.S., the European context might present distinct considerations. Europe’s comparatively stringent regulatory environment and stronger emphasis on ethical AI governance may influence both the perceived opportunities and challenges of AI (15). Against this backdrop, the present study explored stakeholder perspectives on anticipated benefits, concerns, and key challenges. By integrating these insights, the project’s design and deployment can be better aligned with real-world needs and expectations. Moreover, the findings are expected to provide valuable guidance for future research and inform broader applications of AI in healthcare.

## 2 Methods

### 2.1 Study design and participants

Semi-structured interviews were conducted to explore stakeholder perspectives on the benefits, concerns, and challenges associated with developing and implementing the AI-POD tools. Purposive sampling was used to capture diverse viewpoints from individuals engaged in or impacted by AI-POD, alongside experts from social science, ethics, and policy domains (16). Sampling began with individuals affiliated with the AI-POD consortium and was extended through professional networks and open searches of relevant organizations, including obesity associations across European countries. Eligibility criteria included (a) the ability to speak Dutch, French or English, (b) being capable of providing informed consent, and (c) belonging to a relevant stakeholder group. Interviews were conducted between February and July 2025.

### 2.2 Recruitment and data collection

Participants received an informed consent form in advance via email. The form outlined the study’s objectives, methodology, potential benefits and risks, data processing procedures, and data storage protocols. Written informed consent was obtained from all participants prior to their involvement, either in person or electronically. Interviews were scheduled at times and locations convenient for the participants, with the option to conduct interviews remotely via Microsoft Teams. Each interview lasted up to 60 minutes. Prior to the interviews, participants were provided with a concise overview of the AI-POD project, including detailed descriptions of its two primary components: a citizen-facing mobile health application and a clinical decision support system (CDSS). Additionally, a brief introductory video produced by the consortium was made available^1^. All interviews were guided by a semi-structured interview guide (Supplementary file 1).

### 2.3 Sample size and data analysis

A total of 21 interviews were conducted between February and July 2025. All interviews were audio-recorded, transcribed verbatim, and pseudonymized to ensure participant confidentiality. Data were analyzed using inductive content analysis as described by Vears et al., supported by Nvivo (17, 18). Analysis proceeded through three iterative stages: (1) Initial big-picture coding; (2) Second-round coding – developing subcategories and fine-grained codes; (3) Refining the fine-grained subcategories (17). Coding was conducted independently by two researchers (KG and TMF). Differences in interpretation were discussed until consensus was reached, thereby enhancing intercoder reliability and minimizing individual bias. For interviews conducted in Dutch, coding (KG) was verified by a third researcher (EVS), ensuring conceptual consistency across languages.

### 2.4 Ethics statement

This study was reviewed and approved by the Social and Societal Ethics Committee (KU Leuven) (G-2024-7872-R2). All participants provided their written informed consent to participate in this study. Participation was voluntary and participants had the right to withdraw at any stage without consequence. All obtained interview data were pseudonymized. The research was conducted in accordance with the Declaration of Helsinki and local statutory requirements.

## 3 Results

Our study sample consisted of 21 participants, purposively selected to ensure a broad representation of relevant stakeholder groups across the healthcare and innovation landscape. Participants held diverse professional backgrounds, including radiology (*n* = 5), artificial intelligence (*n* = 4), medical informatics and healthcare innovation (*n* = 2), dietetics (*n* = 2), endocrinology (*n* = 2), and general practice (*n* = 1). In addition, our sample included two patient representatives (*n* = 2), as well as individuals with expertise in social sciences and ethics (*n* = 1), law and policy (*n* = 1), and public health (*n* = 1). Most participants were based in Belgium (*n* = 16), with additional representation from Austria (*n* = 3), the United Kingdom (*n* = 1), and Sweden (*n* = 1). Seven participants were affiliated with the AI-POD consortium, while the remaining 14 were external stakeholders. Our study sample included 12 female participants, thereby providing a balanced representation of gender perspectives. An overview of these perspectives is provided in (Table 1).

**Table 1:** Overview of stakeholder perspectives.

(Sub)categories	Important concepts
Perceived benefits	Multimodal data integration Enhanced risk stratification Patient empowerment
Perceived concerns	Psychological burden Weight stigma and framing of obesity Interpretability and communication of risk Equity and accessibility
Development challenges	Algorithmic bias Data integration and quality Limited evidence for personalized interventions
Implementation challenges	Engaging underserved populations Socio-economic determinants Healthcare professional engagement Integration and usability

### 3.1 Perceived benefits

Participants expressed cautious optimism toward the AI-POD tools, reflecting a form of conditional trust in which acceptance depended on the perception that such technologies would complement rather than replace professional expertise. The tools’ strength was seen in their capacity to integrate diverse data sources - including imaging, lifestyle, and clinical parameters –to support longitudinal and individualized care.

*…it (i.e., the AI-POD tools) sounds very novel, and I like the idea of using several sources of data as well and combining them. Because very often you only have clinical data…* – Participant with background in social sciences

Participants particularly valued the system’s potential to enhance risk stratification, enabling clinicians to tailor interventions to patients’ needs. They linked fairness and precision in care delivery, highlighting that more accurate and continuous data could reduce reliance on incomplete or self-biased reports.

*People often hide things in a consultation, or give wrong information and I think the information the physician will get in this way (i.e. through the wearable and citizen-facing health app) will be more accurate.* – Participant with background in radiology

Beyond professional utility, the citizen-facing mobile health application was framed as promoting patient awareness and health literacy. Participants viewed it as a tool to foster self-awareness and motivation, provided it remained embedded within a broader treatment trajectory.

*The app towards the patients, I think it is a nice add-on, towards someone who has obesity and is being followed-up, to have some more control themselves. And, maybe, being confronted with the evolution of their health. I think it (i.e., the citizen-facing mobile health app) is positive, but I think it should be part of a treatment trajectory.* – Participant with background in radiology

### 3.2 Perceived concerns

While participants acknowledged potential benefits of the AI-POD tools, they raised critical concerns related to psychological impact, weight stigma, interpretability, and equitable access.

Some feared that repeated exposure to risk scores could increase anxiety and undermine motivation, particularly without clinician guidance, whereas others suggested that transparency might enhance patient engagement. This highlights the need to balance information transparency with emotional safety through careful, context-sensitive framing.

*I fear that if the result is not good, you will panic and go to your general practitioner or the emergency room. So, you really need to watch out with that. It is very important to relativize the risk scores. And it should also be made clear by your physician that you should not panic if you see a bad score. –* Patient representative

Participants also noted that narrowly focusing on body weight could unintentionally reinforce weight stigma, while framing the tools around holistic health and well-being might validate broader patient concerns and reduce stigma.

*I think it can also decrease stigma. Because the situation now, with patients that have obesity, is that their other health concerns are not taken seriously – or are only taken seriously if they show some sort of commitment to reducing their weight. And I think we, as a society, just have to move away from that focus. So, in a way, if the project is designed correctly, it can also reduce stigma. In the sense that it takes people’s concerns seriously outside of reducing weight and just supports them in their journey whether or not they want to change it.* – Participant with background in social sciences

On top of this, questions were raised about whether patients and clinicians could accurately interpret risk percentages, including distinctions between five-year, ten-year, or lifetime risks and what the added value of providing patients with this risk score itself would be.

*I do not see – if I can be so bold – any patient empowerment in a risk score. Because the patient does not know what it means, if their ten-year risk score increases from two to seven…I think patient empowerment is quite limited unless something changes very specifically in the short term. For example, if you stop eating salt and your blood pressure moves from red to green, that is meaningful. But those complex risk calculators often do the opposite.* – Participant with background in endocrinology

Finally, equitable access was emphasized as essential, with barriers such as limited smartphone availability, device incompatibility, low digital literacy, and language differences requiring attention in design and implementation. Although data privacy concerns were generally minor, risks related to algorithmic opacity and potential misuse (e.g., by insurers) were highlighted. To foster understanding and trust, participants recommended the creation of visual aids and fact sheets tailored to both patients and healthcare professionals.

### 3.3 Development challenges

Participants identified various challenges associated with the development of the AI-POD tools, with a primary concern being the potential of introducing or amplifying algorithmic bias. Several types of potential disparities were noted, including gender bias – given that cardiovascular conditions may present differently in women – as well as ethnic, socio-economic and selection biases. In particular; selection bias was linked to the likelihood that study participants would disproportionately represent health-conscious individuals from higher socio-economic backgrounds, thereby limiting the generalizability of the findings. There was broad agreement that any existing imbalances present in the training data would likely be reproduced and amplified by the algorithm. However, some participants viewed this issue not solely as a limitation, but as an opportunity. If carefully monitored, variations in algorithmic performance across subgroups could provide valuable insights into disease mechanisms.

*You will probably get the higher socio-economic class, who can afford the newest phone, of whom it is known that they are more conscious about their health. So, the risk for cardiovascular diseases will be lower in this group than in a group with a different socio-economic status. So by design, there is a bias in your study. –* Participant with background in medical informatics and health innovation

Next, heterogeneous data formats, fragmented hospital systems, and inconsistent standards were viewed as major barriers to creating unified datasets. Questions were also raised about the reliability of the data feeding into the AI-model. Participants highlighted that wearable devices, while convenient, may not meet clinical-grade standards, raising concerns about data validity. The same concern was raised for self-reported data by patients.

*I think all projects where you are compiling data from different data systems, whether it is within a hospital or, even worse, between hospitals with different laboratory systems- The language is different. It is about the data models or data formats, harmonizing them and then also technically, how do you extract the data from different hospitals? Some people get PDFs or even JPEGs of lab reports. –* Participant with background in radiology.

On top of this, a general lack of public engagement with preventive medicine, particularly among individuals who have not yet experienced a cardiovascular event was noted. Conversely, some participants argued that patients living with obesity may demonstrate a higher degree of intrinsic motivation, having often navigated years of weight- and health-related challenges. Nevertheless, the higher prevalence of obesity among individuals from lower socio-economic backgrounds, who may also have lower levels of health literacy, was seen as a potential obstacle to sustained engagement. To enhance uptake and usability, participants emphasized the importance of involving end-users and stakeholders early in the design process to provide feedback on functionality and experience, alongside clear and transparent communication regarding the purpose, benefits, and expectations of the tools. Where engagement remains limited, alternative approaches, such as leveraging causal AI methods or synthetic data, were suggested to maximize the tool’s effectiveness.

*I wonder to what degree patients- If they already had a cardiovascular event, they will be more motivated to follow the recommendations from the app, than someone who has obesity, but who did not have an event yet or does not know there is a risk. I think it is a bit the same with smoking. You can give as much information and risk scores as you want, but they will keep smoking.* – Participant with background in radiology

Lastly, doubts were expressed regarding the evidence base for highly personalized lifestyle interventions. While general recommendations exist, participants noted that the scientific evidence supporting individualized lifestyle advice remains limited, which could challenge the validity and reliability of the system’s output, especially considering the multifaceted nature of obesity.

*From an evidence-based standpoint, we know a few things about lifestyle, but if we talk about the things we are completely sure of, that’s quite limited…And because of that, it is quite difficult, because in a system like this (i.e. the citizen-facing health application) you want to provide concrete advice, but the more concrete you go, the further away you get from what is actually scientifically proven.* – General practitioner

### 3.4 Implementation challenges

In addition to development challenges, participants identified several key barriers to the effective implementation of the AI-POD tools in clinical practice. A central issue involved engaging disengaged or underserved populations, echoing earlier concerns about selection bias. More specifically, participants noted that digital health tools are often adopted and consistently used primarily by individuals who are already motivated to engage with their health, potentially excluding those who might benefit most.

*We know from that research population (i.e., patients with obesity) that if we use apps, the ones who keep using it are the ones who are the most motivated. So the question is: how can you motivate the other group?* – Participant with background in dietetics

On top of this, some stakeholders warned against overemphasizing individual responsibility, arguing that social determinants—such as food cost and environment—profoundly shape health behavior. This reflects a systems-level understanding of obesity prevention. *We should not individualize everything this much. We are shaped by what is around us: all the food, the unhealthy food, which is still cheaper than fruit and vegetables. So, I think we need to approach it from a more societal perspective.* – Participant with background in public health

Given the preventive focus of the AI-POD tools – where benefits may not be immediately visible –the importance of strategies to support sustained engagement was stressed. Suggested approaches included integrating peer support mechanisms, such as community forums, and ensuring that participation is patient-driven rather than externally imposed. A user-initiated approach, where individuals actively seek support, was seen as more likely to promote long-term engagement.

Next, limited access to smartphones and varying levels of digital literacy were identified as potential exclusionary factors, while poorly designed or overly complex digital interfaces, for both patients and health care professionals, were perceived as likely to hinder use and uptake.

In addition, participants described clinicians as potential gatekeepers of adoption. Integration into established care pathways and endorsement by professional bodies were seen as prerequisites for legitimacy and sustained use as well.

*Health care professionals like the way they normally do it. So changes in standard of care, in clinical guidelines are exceptional. This tool comes out of nowhere. How do we convince them to convince the healthcare professional that what we do is great? Is there sufficient evidence?…If the international community supports this kind of system, then I think you can use that to push.* – Participant with background in law and policy

The tools’ strong emphasis on lifestyle modification was also cited as a potential barrier to clinical adoption. Multiple participants pointed out that in cases where advanced cardiovascular disease is already present, pharmacological treatment is often required to manage risk, and a lifestyle-focused intervention alone may be perceived as insufficient or irrelevant. Some participants further highlighted the importance of integration, suggesting that multiple separate tools for lifestyle monitoring and disease prevention could be confusing.

*Imagine you would have a similar tool for early detection of diabetes in obesity patients. So, you will develop a separate tool, that asks for similar information – what did you eat, how much did you move etc. That’s a bit weird. It is better to integrate everything so the patient only needs one app.* – Patient representative

Lastly, debate arose around the most appropriate point of care for implementation. While many participants considered general practitioners (GP) as the logical integration point, others noted that high-risk individuals may not regularly consult their GP. This underscores the need for interdisciplinary collaboration to ensure that the AI-POD tools reach those patients who stand to benefit most.

## 4 Discussion

Within the AI-POD project, predictive tools are being developed to estimate the risk of obesity-related cardiovascular diseases by integrating clinical, imaging, laboratory and lifestyle data. This study explored how diverse European stakeholders perceive the potential benefits, concerns, and challenges associated with such tools. By examining these perspectives during the development phase, this study provides insights into how early stakeholder engagement can inform the responsible design and implementation of AI-enabled preventive technologies.

### 4.1 Predictive potential and multimodal data integration

Stakeholders generally viewed the AI-POD tools as promising innovations, particularly because of their ability to combine multiple sources of patient data and enhance personalized risk prediction. Physicians in particular valued this multimodal approach, expecting it to improve accuracy for populations often underrepresented in traditional cardiovascular models. However, as several participants stressed, the perceived usefulness of such systems depends on demonstrating the quality and clinical validity of their outputs. Conditional trust was common; participants supported the use of the AI-POD tools only if they complemented rather than replaced clinical expertise. Consistent with the Technology Acceptance Model (TAM), clinicians expressed willingness to adopt the tools if they improved diagnostic confidence and workflow efficiency, but hesitancy if the tools lacked transparency or interpretability (19). The challenge of balancing predictive complexity and explainability—also well-documented in AI healthcare research—was thus seen as central to building and maintaining user trust.

Participants also identified practical issues in the collection of lifestyle data. App-based dietary assessment was perceived as more accurate than traditional recall methods, aligning with prior findings by Chmurzynska et al. (20). However the need for frequent manual input reduced perceived ease of use and risked limiting user engagement. To mitigate this, participants suggested the use of image-based food recognition or supermarket data, though both options face technical and ethical limitations (21, 22).

### 4.2 Personalized risk communication

Participants believed that personalized risk information could enhance health awareness and promote health behavior change, though evidence suggests these effects are modest (23). However, several stakeholders also warned that repeated exposure to high-risk feedback might heighten anxiety, leading to disengagement. This ambivalence illustrates how perceived usefulness can be undermined when tools fail to support users emotionally, even if technically sound.

Selecting an appropriate risk communication strategy will therefore be critical for the successful implementation of the AI-POD tools. Both the content and format of delivery can influence how individuals interpret and respond to risk information, shaping their level of engagement, comprehension, and potential for behavior change. Various communication strategies have been explored in the literature, including numerical and graphical representations, interactive tools such as avatars or games, and the use of cardiovascular imaging (24). Evidence suggests that visual formats, particularly those involving imaging results, may be more impactful than numerical estimates, as they provide a more tangible representation of disease risk. For example, showing patients actual images or imaging-derived scores can make the risk feel more immediate and personally relevant (24).

As highlighted by Schulberg et al., both individual factors and the involvement of a healthcare professional are likely to influence the effectiveness of risk communication strategies, supporting the views expressed by participants in our study (24). Schulberg et al. also noted that individuals are generally less engaged in risk discussions when no physical symptoms are present, particularly in the context of cardiovascular disease (24). Notably, their review did not address how obesity may affect the perceived impact of different risk communication methods. Given the unique clinical and psychosocial characteristics of individuals living with obesity, further research to identify the most impactful methods for communicating risk within this population would be of great value.

### 4.3 Digital access and health literacy

Several participants raised concerns about equitable access, highlighting that low digital literacy and socioeconomic barriers could limit adoption of the AI-POD tools. While smartphone ownership continues to rise globally, gaps in digital engagement persist, particularly among groups with obesity who may also experience lower socioeconomic status and health literacy (25–29). Participants emphasized that such disparities affect both the perceived ease of use and actual ability to engage with the tools. These findings align with the TAM framework, which posits that user self-efficacy strongly influences adoption (19). In this case, smartphone self-efficacy—the confidence in using digital health apps—emerged as a major determinant of willingness to use the citizen-facing tool. Participants suggested that the high input burden and lack of immediate, tangible benefits may discourage users, particularly when the preventive nature of the intervention delays visible outcomes. Developers could enhance engagement by simplifying user interfaces, integrating automated data capture, and adding motivational or gamified elements that increase perceived ease of use and provide short-term feedback.

However, improving digital health literacy remains a priority. As Barbati et al. showed, tailored literacy interventions can increase confidence and competence in using digital tools (30). Policymakers should therefore invest in digital health education programs targeting at-risk groups to ensure equitable access to AI-enabled preventive care.

### 4.4 Clinical integration and sustained engagement

Maintaining long-term engagement with health applications continues to be a significant challenge, with usage often declining over time despite promising initial uptake (31). Our findings emphasize that successful implementation requires both sustained patient use and integration into clinical workflows. Aligning the AI-POD tools with existing clinical practices, minimizing duplication of effort, and co-creating solutions with patients and healthcare professionals were highlighted as critical strategies to enhance adoption and relevance. Before education and engagement strategies can be implemented, access pathways must first be identified. Several participants suggested that one effective approach could be to implement the AI-POD tools at the level of the general practitioner (GP). GPs often serve as the primary point of contact for individuals, which may make it more feasible to reach and engage these groups through existing care structures. Moreover, existing literature highlights the importance of co-creation with patients and other end-users as a strategy to enhance adoption and relevance of digital health tools (32).

### 4.5 Implications and future directions

Our findings offer important insights for the refinement and responsible implementation of the AI-POD tools in clinical practice. Critical considerations include enhancing predictive performance while preserving model explainability, reducing user burden, designing effective risk communication strategies, addressing disparities in digital access and health literacy, and ensuring seamless integration within existing healthcare systems, aligning with the TAM model (19). Future research should focus on developing tailored communication approaches for individuals living with obesity and identifying strategies to promote sustained engagement with AI-based health interventions. Finally, our findings underscore the importance of embedding co-creation as a formal requirement within EU policy on AI and health innovation. Early and continuous stakeholder involvement allows potential challenges to be identified and mitigated in advance, improving user alignment and cost-efficiency. By ensuring that technologies are designed with and for end users, such policies would enhance the likelihood of successful implementation, adoption, and equitable benefit realization across European healthcare systems.

### 4.6 Limitations

Despite its strengths, this study also has several limitations. First, although efforts were made to engage stakeholders from multiple European countries, recruitment primarily relied on the professional networks of the research team and consortium partners, resulting in a predominance of participants based in Belgium. Second, while the study aimed to include a wide range of perspectives, certain stakeholder groups—such as psychologists—were not represented, which may have limited the breadth of insights obtained. Language further posed a constraint, as interviews were conducted only in Dutch, French, or English, potentially hindering participation from individuals less proficient in these languages. Third, although the interviews provided a diverse and informative dataset, the study was exploratory rather than exhaustive and did not aim to reach full data saturation. As such, the findings should be interpreted as indicative rather than representative of all stakeholder perspectives. In addition, while the inclusion of multiple coders helped to enhance analytical rigor and minimize bias, variations in linguistic accessibility meant that only the English interviews were double-coded, with Dutch transcripts reviewed separately for consistency. Finally, a substantial proportion of interviewees had backgrounds in radiology or AI development and did not practice clinically, which may have limited the depth of insights into real-world clinical implementation of the AI-POD tools. Future research could expand participation across additional professional and national contexts to further validate and extend these findings.

## Data Availability

The datasets presented in this article are not readily available because it is not possible to fully anonymize the interview data. Requests to access the datasets should be directed to kaat.goossens@kuleuven.be.

## Footnotes

1
https://www.youtube.com/watch?v=fVA_qe5VAUw&t=128s
