# Supplementary material for: AI-driven tools for the prediction of obesity-related vascular diseases: stakeholder perspectives and challenges
Source: Front Public Health. 2025 Dec 4;13:1713882. doi: 10.3389/fpubh.2025.1713882 (PMC12711769; doi:10.3389/fpubh.2025.1713882)
Supplement: Supplementary file 1 [file Table_1.docx]

**Supplementary file 1: interview guide**

Introduction:

- Overview of important elements from the informed consent form (e.g. audio- and/or video-recording, right to withdraw, voluntary participation…)
- Introduction on the AI-POD project: verbal or video (<https://www.youtube.com/watch?v=fVA_qe5VAUw&t=128s>)

Introductory questions:

- AI:
- Personal definition of AI
- Use of AI in daily / professional life
- Health monitoring:
- Personal use of health monitoring
- Perception of health monitoring experience of patients with obesity

Key questions:

- AI-POD tools:
  - Overall attitude towards the AI-POD tools
  - Attitude towards / perspectives on risk scoring in the population of patients with obesity
    - Advantages / benefits ⬄ disadvantages
    - Concerns
  - Other benefits
    - AI-POD clinical decision support system (CDSS)
    - AI-POD citizen-facing mobile health application
  - Other concerns
    - AI-POD clinical decision support system (CDSS)
    - AI-POD citizen-facing mobile health application
  - Development challenges
    - AI-POD clinical decision support system (CDSS)
    - AI-POD citizen-facing mobile health application
      - Probing questions: bias? data quality / quantity?...
  - Implementation challenges
    - AI-POD clinical decision support system (CDSS)
    - AI-POD citizen-facing mobile health application
      - Probing questions: adoption? access?

Closing questions:

- Most important message
- Anything not discussed?
